# Supplementary material for: A Cohort Study of Korean Radiation Workers: Baseline Characteristics of Participants
Source: Int J Environ Res Public Health. 2020 Mar 30;17(7):2328. doi: 10.3390/ijerph17072328 (PMC7177891; doi:10.3390/ijerph17072328)
Supplement: Supplementary file 1 [file ijerph-17-02328-s001.zip › ijerph-736979-supplementary.pdf]

Supplemental Table S1. Demographic characteristics and occupational history by occupation.

	Public Institute (n = 676)	Education Institute (n = 2010)	Military (n = 165)	Industrial Radiography (n = 3517)	Industry (n = 3886)	Research Institute (n = 1139)	Nuclear Power Plant (n = 6328)	Medical Institute (n = 2887)
**Demographic characteristics**								
Sex								
Men	592 (87.6%)	1257 (62.5%)	153 (92.7%)	3397 (96.6%)	3709 (95.4%)	932 (81.8%)	6146 (97.1%)	1645 (57.0%)
Women	84 (12.4%)	753 (37.5%)	12 (7.3%)	120 (3.4%)	177 (4.6%)	207 (18.2%)	182 (2.9%)	1242 (43.0%)
Birth year								
~1960	49 (7.2%)	60 (3.0%)	1 (0.6%)	93 (2.6%)	184 (4.7%)	182 (16.0%)	681 (10.8%)	141 (4.9%)
1961-1970	146 (21.6%)	149 (7.4%)	16 (9.7%)	309 (8.8%)	783 (20.1%)	210 (18.4%)	1364 (21.6%)	472 (16.3%)
1971-1980	160 (23.7%)	309 (15.4%)	41 (24.8%)	1174 (33.4%)	1240 (31.9%)	290 (25.5%)	1865 (29.5%)	812 (28.1%)
1981~	321 (47.5%)	1492 (74.2%)	107 (64.8%)	1941 (55.2%)	1679 (43.2%)	457 (40.1%)	2418 (38.2%)	1462 (50.6%)
Education level								
Less than high school graduation	4 (0.6%)	0 (0.0%)	0 (0.0%)	15 (0.4%)	21 (0.6%)	0 (0.0%)	113 (1.8%)	6 (0.2%)
High school graduation	132 (19.8%)	240 (12.5%)	45 (28.3%)	1215 (35.5%)	1321 (35.0%)	28 (2.5%)	1504 (24.2%)	54 (1.9%)
College graduation and above	532 (79.6%)	1682 (87.5%)	114 (71.7%)	2197 (64.1%)	2431 (64.4%)	1101 (97.5%)	4602 (74.0%)	2743 (97.9%)
Marital status								
Unmarried	237 (35.5%)	1293 (67.1%)	75 (46.9%)	1789 (52.1%)	1146 (30.4%)	343 (30.4%)	1948 (31.4%)	1050 (37.5%)
Married/living together	425 (63.6%)	626 (32.5%)	83 (51.9%)	1604 (46.7%)	2590 (68.6%)	778 (69.0%)	4138 (66.7%)	1737 (62.0%)
Other (divorced, widow, separated)	6 (0.9%)	9 (0.5%)	2 (1.3%)	42 (1.2%)	37 (1.0%)	7 (0.6%)	116 (1.9%)	14 (0.5%)
BMI, kg/m2								
Underweight (<18.5)	21 (3.3%)	104 (6.0%)	0 (0.0%)	64 (1.9%)	40 (1.1%)	39 (3.7%)	77 (1.3%)	145 (5.6%)
Normal weight (18.5–24.9)	431 (66.8%)	1173 (67.5%)	101 (65.6%)	1756 (53.3%)	2087 (58.0%)	681 (64.5%)	3633 (60.5%)	1815 (69.9%)
Overweight (25.0–29.9)	175 (27.1%)	394 (22.7%)	46 (29.9%)	1211 (36.7%)	1294 (35.9%)	299 (28.3%)	2019 (33.6%)	574 (22.1%)
Obese (≥30.0)	18 (2.8%)	67 (3.9%)	7 (4.5%)	266 (8.1%)	180 (5.0%)	36 (3.4%)	275 (4.6%)	64 (2.5%)
Regular exercise								
No	270 (40.2%)	1059 (53.1%)	24 (14.5%)	1847 (53.0%)	1633 (42.4%)	461 (40.8%)	2387 (38.0%)	1380 (48.1%)
Yes	402 (59.8%)	934 (46.9%)	141 (85.5%)	1641 (47.0%)	2215 (57.6%)	670 (59.2%)	3890 (62.0%)	1492 (51.9%)
Smoking status								
Never (non-smoker)	279 (41.7%)	1364 (68.5%)	90 (54.5%)	893 (25.6%)	1274 (33.1%)	559 (49.5%)	2079 (33.2%)	1904 (66.4%)
Ex-smoker	133 (19.9%)	192 (9.6%)	20 (12.1%)	565 (16.2%)	802 (20.8%)	217 (19.2%)	1303 (20.8%)	383 (13.4%)

	Public Institute (n = 676)		Education Institute (n = 2010)		Military (n = 165)		Industrial Radiography (n = 3517)		Industry (n = 3886)		Research Institute (n = 1139)		Nuclear Power Plant (n = 6328)		Medical Institute (n = 2887)	
Yes (smoker)	257	(38.4%)	434	(21.8%)	55	(33.3%)	2029	(58.2%)	1776	(46.1%)	353	(31.3%)	2878	(46.0%)	579	(20.2%)
Alcohol status																
No	108	(16.0%)	381	(19.1%)	23	(13.9%)	528	(15.1%)	517	(13.4%)	208	(18.3%)	895	(14.2%)	616	(21.4%)
Yes	567	(84.0%)	1616	(80.9%)	142	(86.1%)	2972	(84.9%)	3346	(86.6%)	929	(81.7%)	5403	(85.8%)	2264	(78.6%)
Experience with thyroid cancer screening (thyroid ultrasound)																
Never	524	(83.0%)	1538	(86.0%)	143	(87.7%)	3164	(94.2%)	2551	(71.6%)	641	(60.6%)	3424	(58.5%)	2115	(77.8%)
1 time	71	(11.3%)	157	(8.8%)	9	(5.5%)	139	(4.1%)	555	(15.6%)	200	(18.9%)	1115	(19.1%)	364	(13.4%)
2 times	15	(2.4%)	40	(2.2%)	6	(3.7%)	31	(0.9%)	181	(5.1%)	64	(6.1%)	491	(8.4%)	128	(4.7%)
3 times	21	(3.3%)	54	(3.0%)	5	(3.1%)	26	(0.8%)	274	(7.7%)	152	(14.4%)	824	(14.1%)	110	(4.1%)
**Occupational history**																
Calendar year of hiring																
~1989	75	(11.1%)	6	(0.3%)	0	(0.0%)	173	(4.9%)	98	(2.5%)	163	(14.3%)	630	(10.0%)	127	(4.4%)
1990–1999	93	(13.8%)	108	(5.4%)	5	(3.0%)	479	(13.6%)	410	(10.6%)	143	(12.6%)	1133	(17.9%)	323	(11.2%)
2000–2009	114	(16.9%)	274	(13.6%)	60	(36.4%)	1006	(28.6%)	823	(21.2%)	270	(23.7%)	1293	(20.4%)	729	(25.3%)
2010~	394	(58.3%)	1622	(80.7%)	100	(60.6%)	1859	(52.9%)	2555	(65.7%)	563	(49.4%)	3272	(51.7%)	1708	(59.2%)
Age at the started of radiation work																
<20	4	(0.6%)	68	(3.4%)	23	(13.9%)	277	(7.9%)	44	(1.1%)	0	(0.0%)	363	(5.7%)	6	(0.2%)
20–29	444	(65.7%)	1471	(73.2%)	90	(54.5%)	2430	(69.1%)	1683	(43.3%)	573	(50.3%)	3619	(57.2%)	1858	(64.4%)
30–39	152	(22.5%)	322	(16.0%)	35	(21.2%)	738	(21.0%)	1489	(38.3%)	462	(40.6%)	1553	(24.5%)	698	(24.2%)
40–49	54	(8.0%)	112	(5.6%)	15	(9.1%)	54	(1.5%)	511	(13.1%)	86	(7.6%)	491	(7.8%)	242	(8.4%)
≥50	22	(3.3%)	37	(1.8%)	2	(1.2%)	18	(0.5%)	159	(4.1%)	18	(1.6%)	302	(4.8%)	83	(2.9%)
Employment status																
Regular employment	567	(84.8%)	695	(43.7%)	124	(75.6%)	3397	(98.5%)	3685	(95.6%)	781	(69.8%)	4973	(79.9%)	2228	(77.9%)
Irregular employment (temporary contract)	97	(14.5%)	863	(54.3%)	40	(24.4%)	48	(1.4%)	150	(3.9%)	327	(29.2%)	743	(11.9%)	617	(21.6%)
Irregular employment (daily contract)	5	(0.7%)	32	(2.0%)	0	(0.0%)	3	(0.1%)	18	(0.5%)	11	(1.0%)	505	(8.1%)	15	(0.5%)
Duration of employment, years																
≤4	308	(45.6%)	1281	(63.7%)	78	(47.3%)	1294	(36.8%)	2062	(53.1%)	446	(39.2%)	2485	(39.3%)	1311	(45.4%)
5–9	137	(20.3%)	432	(21.5%)	42	(25.5%)	904	(25.7%)	758	(19.5%)	211	(18.5%)	1198	(18.9%)	628	(21.8%)
10–14	51	(7.5%)	143	(7.1%)	38	(23.0%)	534	(15.2%)	425	(10.9%)	133	(11.7%)	698	(11.0%)	350	(12.1%)
≥15 years	180	(26.6%)	154	(7.7%)	7	(4.2%)	785	(22.3%)	641	(16.5%)	349	(30.6%)	1947	(30.8%)	598	(20.7%)

	Public Institute (n = 676)		Education Institute (n = 2010)		Military (n = 165)		Industrial Radiography (n = 3517)		Industry (n = 3886)		Research Institute (n = 1139)		Nuclear Power Plant (n = 6328)		Medical Institute (n = 2887)	
Experience of warning for exceeding 5mSv per quarter																
No	611	(92.3%)	1659	(92.8%)	157	(96.3%)	2970	(88.5%)	3338	(87.8%)	1066	(95.3%)	5417	(90.0%)	2639	(93.1%)
Yes	27	(4.1%)	15	(0.8%)	3	(1.8%)	283	(8.4%)	111	(2.9%)	19	(1.7%)	369	(6.1%)	74	(2.6%)
I don't know	24	(3.6%)	113	(6.3%)	3	(1.8%)	103	(3.1%)	353	(9.3%)	33	(3.0%)	235	(3.9%)	123	(4.3%)
Night shifts																
None	422	(62.9%)	1639	(83.8%)	115	(71.4%)	922	(26.6%)	1630	(42.5%)	938	(83.0%)	2949	(47.5%)	1746	(61.2%)
<1 year	37	(5.5%)	122	(6.2%)	18	(11.2%)	746	(21.5%)	239	(6.2%)	70	(6.2%)	1103	(17.8%)	209	(7.3%)
1–5 years	121	(18.0%)	154	(7.9%)	15	(9.3%)	1439	(41.5%)	649	(16.9%)	80	(7.1%)	1236	(19.9%)	521	(18.3%)
>5 years	91	(13.6%)	42	(2.1%)	13	(8.1%)	357	(10.3%)	1313	(34.3%)	42	(3.7%)	919	(14.8%)	375	(13.2%)
Radiation source																
None	137	(20.9%)	175	(9.8%)	2	(1.2%)	70	(2.0%)	139	(3.7%)	144	(12.7%)	2076	(36.3%)	130	(4.5%)
Sealed isotope	123	(18.8%)	270	(15.1%)	21	(12.8%)	2408	(68.1%)	1262	(33.2%)	200	(17.6%)	508	(8.9%)	517	(17.8%)
Unsealed isotope	166	(25.3%)	478	(26.7%)	1	(0.6%)	36	(1.0%)	232	(6.1%)	339	(29.8%)	274	(4.8%)	904	(31.1%)
Radiation-generating device	128	(19.5%)	723	(40.4%)	133	(81.1%)	909	(25.7%)	1566	(41.2%)	369	(32.5%)	629	(11.0%)	1102	(37.9%)
Not sure	102	(15.5%)	142	(7.9%)	7	(4.3%)	111	(3.1%)	604	(15.9%)	85	(7.5%)	2237	(39.1%)	251	(8.6%)
Distance from radiation source																
<1 m	250	(39.2%)	691	(40.8%)	6	(3.8%)	41	(1.3%)	754	(20.4%)	347	(32.4%)	757	(13.7%)	906	(33.2%)
1–3 m	186	(29.2%)	488	(28.8%)	22	(13.8%)	266	(8.1%)	1510	(40.8%)	321	(30.0%)	1347	(24.5%)	726	(26.6%)
>3 m	202	(31.7%)	513	(30.3%)	132	(82.5%)	2968	(90.6%)	1434	(38.8%)	403	(37.6%)	3403	(61.8%)	1094	(40.1%)
While engaged in radiation work, white blood cell counts fell below the normal range																
No	611	(92.2%)	1616	(89.7%)	155	(94.5%)	3138	(93.1%)	3354	(88.1%)	1038	(93.1%)	5382	(89.2%)	2628	(92.5%)
Yes	17	(2.6%)	36	(2.0%)	4	(2.4%)	107	(3.2%)	61	(1.6%)	26	(2.3%)	172	(2.8%)	75	(2.6%)
Not sure (or had never had a health examination.)	35	(5.3%)	149	(8.3%)	5	(3.0%)	126	(3.7%)	393	(10.3%)	51	(4.6%)	483	(8.0%)	139	(4.9%)

BMI, body mass index.

Supplemental Table S2. Experience of medical radiation-related examination or procedure for diagnosis or treatment in the last 3 years.

	Public Institute (n = 676)	Education Institute (n = 2010)	Military (n = 165)	Industrial Radiography (n = 3517)	Industry (n = 3886)	Research Institute (n = 1139)	Nuclear Power Plant (n = 6328)	Medical Institute (n = 2887)	Total (N = 20,608)
Standard X-ray imaging (chest, abdomen, head, transcranial, or limbs)									
Never	74 (11.1%)	366 (18.8%)	28 (17.2%)	533 (15.6%)	554 (14.6%)	106 (9.4%)	911 (14.8%)	212 (7.4%)	2784 (13.8%)
1 time	142 (21.3%)	494 (25.3%)	23 (14.1%)	564 (16.5%)	794 (21.0%)	195 (17.4%)	1304 (21.2%)	515 (18.0%)	4031 (20.0%)
2 times	107 (16.0%)	420 (21.5%)	18 (11.0%)	537 (15.7%)	649 (17.2%)	172 (15.3%)	1083 (17.6%)	456 (15.9%)	3442 (17.1%)
≥ 3 times	344 (51.6%)	669 (34.3%)	94 (57.7%)	1792 (52.3%)	1786 (47.2%)	650 (57.9%)	2857 (46.4%)	1678 (58.7%)	9870 (49.0%)
Interoral radiography or panoramic radiography (face and mouth)									
Never	301 (46.9%)	1228 (64.0%)	90 (58.1%)	2103 (63.8%)	2391 (66.9%)	633 (58.4%)	3950 (67.4%)	1568 (56.8%)	12,264 (63.6%)
1 time	133 (20.7%)	424 (22.1%)	30 (19.4%)	706 (21.4%)	714 (20.0%)	262 (24.2%)	1154 (19.7%)	724 (26.2%)	4147 (21.5%)
2 times	83 (12.9%)	166 (8.7%)	17 (11.0%)	279 (8.5%)	294 (8.2%)	112 (10.3%)	469 (8.0%)	300 (10.9%)	1720 (8.9%)
≥ 3 times	125 (19.5%)	101 (5.3%)	18 (11.6%)	210 (6.4%)	177 (4.9%)	77 (7.1%)	291 (5.0%)	167 (6.1%)	1166 (6.0%)
Computed tomography (CT)									
Never	436 (69.8%)	1577 (82.5%)	133 (85.8%)	2737 (83.6%)	2551 (71.3%)	772 (71.0%)	4298 (73.3%)	2133 (77.6%)	14,637 (76.1%)
1 time	148 (23.7%)	264 (13.8%)	17 (11.0%)	398 (12.2%)	716 (20.0%)	239 (22.0%)	1174 (20.0%)	469 (17.1%)	3425 (17.8%)
2 times	22 (3.5%)	48 (2.5%)	4 (2.6%)	87 (2.7%)	191 (5.3%)	44 (4.0%)	260 (4.4%)	103 (3.7%)	759 (3.9%)
≥ 3 times	19 (3.0%)	22 (1.2%)	1 (0.6%)	52 (1.6%)	118 (3.3%)	33 (3.0%)	131 (2.2%)	43 (1.6%)	419 (2.2%)
Fluoroscopy									
Never	579 (95.4%)	1853 (98.1%)	150 (99.3%)	3142 (97.1%)	3309 (95.5%)	1009 (95.4%)	5457 (95.3%)	2654 (97.9%)	18,153 (96.2%)
1 time	21 (3.5%)	28 (1.5%)	1 (0.7%)	42 (1.3%)	109 (3.1%)	37 (3.5%)	204 (3.6%)	42 (1.5%)	484 (2.6%)
2 times	1 (0.2%)	5 (0.3%)	0 (0.0%)	18 (0.6%)	24 (0.7%)	7 (0.7%)	36 (0.6%)	13 (0.5%)	104 (0.6%)
≥ 3 times	6 (1.0%)	3 (0.2%)	0 (0.0%)	33 (1.0%)	23 (0.7%)	5 (0.5%)	31 (0.5%)	3 (0.1%)	104 (0.6%)
Oral or intervenous administration of radioactive medicines for radiological medical examinations or treatment									
Never	577 (94.6%)	1826 (97.1%)	148 (98.0%)	2066 (97.5%)	3259 (94.2%)	967 (90.7%)	5308 (93.5%)	2562 (94.4%)	16,713 (94.5%)
1 time	27 (4.4%)	49 (2.6%)	1 (0.7%)	31 (1.5%)	145 (4.2%)	85 (8.0%)	302 (5.3%)	128 (4.7%)	768 (4.3%)
2 times	3 (0.5%)	5 (0.3%)	1 (0.7%)	5 (0.2%)	33 (1.0%)	9 (0.8%)	33 (0.6%)	18 (0.7%)	107 (0.6%)
≥ 3 times	3 (0.5%)	1 (0.1%)	1 (0.7%)	18 (0.8%)	24 (0.7%)	5 (0.5%)	37 (0.7%)	7 (0.3%)	96 (0.6%)
Breast X-ray imaging (mammography for breast cancer diagnosis)									
Never	569 (94.4%)	1758 (92.5%)	147 (99.3%)	3151 (98.5%)	3287 (96.9%)	956 (91.7%)	5528 (98.1%)	2375 (86.9%)	17,771 (95.3%)
1 time	19 (3.2%)	93 (4.9%)	1 (0.7%)	24 (0.8%)	57 (1.7%)	41 (3.9%)	74 (1.3%)	216 (7.9%)	525 (2.8%)
2 times	7 (1.2%)	32 (1.7%)	0 (0.0%)	5 (0.2%)	25 (0.7%)	22 (2.1%)	14 (0.2%)	77 (2.8%)	182 (1.0%)
≥ 3 times	8 (1.3%)	17 (0.9%)	0 (0.0%)	18 (0.6%)	22 (0.6%)	24 (2.3%)	18 (0.3%)	64 (2.3%)	171 (0.9%)
Interventional radiography or angiography									
Never	594 (98.3%)	1874 (98.9%)	149 (99.3%)	3196 (98.7%)	3396 (98.0%)	1028 (97.4%)	5556 (97.3%)	2690 (99.1%)	18,483 (98.2%)

	Public Institute (n = 676)		Education Institute (n = 2010)		Military (n = 165)		Industrial Radiography (n = 3517)		Industry (n = 3886)		Research Institute (n = 1139)		Nuclear Power Plant (n = 6328)		Medical Institute (n = 2887)		Total (N = 20,608)	
1 time	8	(1.3%)	17	(0.9%)	0	(0.0%)	16	(0.5%)	46	(1.3%)	24	(2.3%)	123	(2.2%)	19	(0.7%)	253	(1.3%)
2 times	1	(0.2%)	2	(0.1%)	1	(0.7%)	7	(0.2%)	12	(0.3%)	3	(0.3%)	13	(0.2%)	3	(0.1%)	42	(0.2%)
≥ 3 times	1	(0.2%)	1	(0.1%)	0	(0.0%)	19	(0.6%)	10	(0.3%)	0	(0.0%)	16	(0.3%)	3	(0.1%)	50	(0.3%)
Radiation theraphy (External radiation therapy or internal radiation therapy)																		
Never	596	(98.3%)	1885	(99.4%)	150	(100.0%)	3196	(98.7%)	3414	(98.4%)	1051	(99.2%)	5626	(98.5%)	2703	(99.6%)	15,425	(98.9%)
1 time	7	(1.2%)	8	(0.4%)	0	(0.0%)	19	(0.6%)	34	(1.0%)	6	(0.6%)	58	(1.0%)	7	(0.3%)	120	(0.8%)
2 times	1	(0.2%)	2	(0.1%)	0	(0.0%)	5	(0.2%)	8	(0.2%)	1	(0.1%)	10	(0.2%)	1	(0.0%)	23	(0.1%)
≥ 3 times	2	(0.3%)	1	(0.1%)	0	(0.0%)	19	(0.6%)	12	(0.3%)	1	(0.1%)	16	(0.3%)	2	(0.1%)	34	(0.2%)

Supplmental Table S3. Characteristics of exposed group and non-exposed group(≤0.1mSv) in the cohort.

Characteristics	Exposed Group (n = 12,065)		Non-Exposed Group (n = 7462)	
Person-years				
Sum	126,366.41		32,448.97	
Mean±SD	10.47±6.14		4.35±4.40	
Median	10.00		3.00	
Sex				
Men	11,082	(91.9%)	5861	(78.5%)
Women	983	(8.1%)	1601	(21.5%)
Birth year				
~1950	78	(0.7%)	25	(0.3%)
1950–1959	895	(7.4%)	346	(4.6%)
1960–1969	2506	(20.8%)	844	(11.3%)
1970–1979	4080	(33.8%)	1639	(22.0%)
1980–1989	3929	(32.6%)	3385	(45.4%)
1990~	577	(4.8%)	1223	(16.4%)
Cumulative radiation dose (mSv)				
Mean±SD	14.48±28.80			
Smoking status, n(%)				
Non-smoker	4036	(33.7%)	3874	(52.4%)
Ex-smoker	2395	(20.0%)	1092	(14.8%)
Smoker	5533	(46.3%)	2429	(32.9%)
Occupation, n(%)				
Public institute	205	(2.8%)	439	(3.6%)
Education institute	1288	(17.3%)	517	(4.3%)
Military	80	(1.1%)	84	(0.7%)
Industrial radiography	232	(3.1%)	3183	(26.4%)
Industry	2178	(29.2%)	1467	(12.2%)
Research institute	633	(8.5%)	438	(3.6%)
Nuclear power plant	1956	(26.2%)	4081	(33.9%)
Medical institute	890	(11.9%)	1856	(15.4%)
